# Plackett-Burman Design and Response Surface Optimization of Medium Trace Nutrients for Glycolipopeptide Biosurfactant Production

**DOI:** 10.18869/acadpub.ibj.21.4.249

**Published:** 2017-07

**Authors:** Maurice George Ekpenyong, Sylvester Peter Antai, Atim David Asitok, Bassey Offiong Ekpo

**Affiliations:** 1 Environmental Microbiology and Biotechnology Unit, Department of Microbiology, Faculty of Biological Sciences, University of Calabar, P.M.B.1115 Calabar, Nigeria; 2 Exploration, Research and Services Section, Research and Development (R&D) Division, Nigerian National Petroleum Corporation (NNPC), Port-Harcourt, Nigeria; 3 Environmental Geochemistry Unit, Department of Pure and Applied Chemistry, Faculty of Physical Sciences, University of Calabar, P.M.B. 1115, Calabar, Nigeria

**Keywords:** *Pseudomonas aeruginosa*, Surface-active agents, Fermentation, Nickel, Copper

## Abstract

**Background:**

A glycolipopeptide biosurfactant produced by *Pseudomonas aeruginosa* strain IKW1 reduced the surface tension of fermentation broth from 71.31 to 24.62 dynes/cm at a critical micelle concentration of 20.80 mg/L. The compound proved suitable for applications in emulsion stabilization in food, as well as in cosmetic and pharmaceutical formulations.

**Methods:**

In the present study, Plackett-Burman design (PBD) and response surface method (RSM) were employed to screen and optimize concentrations of trace nutrients in the fermentation medium, to increase surfactant yield.

**Results:**

The PBD selected 5 significant trace nutrients out of the 12 screened. The RSM, on the other hand, resulted in the production of 84.44 g glycolipopeptide/L in the optimized medium containing 1.25 mg/L nickel, 0.125 mg/L zinc, 0.075 mg/L iron, 0.0104 mg/L boron, and 0.025 mg/L copper.

**Conclusion:**

Significant second-order quadratic models for biomass (*P*<0.05; adjusted *R*^2^=94.29%) and biosurfactant (*R*^2^=99.44%) responses suggest excellent goodness-of-fit of the models. However, their respective non-significant lack-of-fit (Biomass: *F*=1.28; *P*=0.418; Biosurfactant: *F*=1.20; *P*=0.446) test results indicate their adequacy to explain data variations in the experimental region. The glycolipopeptide is recommended for the formulation of inexpensive pharmaceutical products that require surface-active compounds.

## INTRODUCTION

Biosurfactants are amphiphilic compounds that reduce the free surface enthalpy per unit area of surfaces and interfaces[1]. They are derived from biological sources including plants, animals, and microorganisms; however, world commercial interest has focused, principally, on microbial derivatives from bacteria, yeasts, and molds[2].

Microbial surfactants are of diverse chemical nature, including glycolipids[3], flavolipids[4], lipopeptides[5], and glycolipopeptides[6-8]. Each biosurfactant performs specialized function(s) in their respective producing organisms. Such functions include solubilization, emulsification, pathogenesis, antibiosis, swarming motility, as well as wetting of and attachment to surfaces[9].

Commercial interest in biosurfactant production has heightened over the years owing to their increasingly widening applications. Surface-active compounds are applied for different purposes in pharmaceutical, detergent, food, cosmetic, petroleum, and agricultural sectors, as well as the environment[10,11]. A threatening bottleneck in microbial production of biosurfactants is the economics of production[1]. Several strategies exist to increase biosurfactant yield, that include the use of low-cost substrates, medium optimization, strain improvement, and development of fermentation conditions[12,13].

Optimization of fermentation media involving major and trace nutrients, as well as environmental and fermentation conditions has been documented[14-16]. A good number of biosurfactant fermentation media compositions incorporating both major and minor nutrients have been reported without information on source of trace element composition. A remarkable study on trace mineral composition of a biosurfactant medium has been reported by Joshi *et al*.[17].

Trace elements are required in small amounts and play essential roles in cellular metabolism, mostly as enzyme co-factors and/or side groups in some microbial metabolites[18]. Selection of significant trace elements for incorporation into fermentation media is an uphill task from the viewpoint of variable size. A classical method for screening large variables is the use of Plackett-Burman design (PBD). It is a small-sized two-level factorial experimental design programmed to identify critical physicochemical parameters from *N* number of variables in *N+*1experiments without recourse to the interaction effects between and among the variables. Since the sample size is traditionally small, the interaction effects are completely shrouded in the main effects. PBD therefore simply screens the design space to detect large main effects[19]. The selected parameters are further optimized by the means of an appropriate design technique of a response surface method (RSM). This method is a collection of statistical techniques that uses design of experiments to build models, evaluate the effect of factors and predict optimum conditions for the factors[20]. The objective of the present study was to optimize the conditions of trace nutrients in a fermentation medium using PBD and RSM to improve biosurfactant yield.

## MATERIALS AND METHODS

### Producing organism

The bacterium, *Pseudomonas aeruginosa* strain IKW1, was earlier isolated[6,21]. The stock culture of the bacterium was retrieved and subcultured in Tryptic Soy Agar by the quadrant-streak plate technique at 30°C for 36 h. The bacterium was passed a second time through the reactivation step in the same medium for 24 h, after which a loop-full of culture was used to inoculate freshly prepared Luria broth in 50-mL Erlenmeyer flask containing 10 mL of medium. Flasks were incubated on a rotary shaker at 150 rpm at room temperature (28±2°C) for 18 h.

### Optimization experiments

#### Screening trace elements

The PBD incorporated into MINITAB 17 statistical software (trial version) was used to screen 12 trace nutrients in 20 randomized experimental runs. The nutrients were purchased from Sigma Aldrich, USA and included NaCl, KCl, CaCl_2_, MgSO_4_.7H_2_O, CuSO_4_.5H_2_O, NiCl_2_.6H2O, FeCl_3_, ZnCl_2_.7H_2_O, K_3_BO_3_, MoNa_2_O_4_.2H_2_O, CoCl_2_, and MnSO_4_.4H_2_O. Each was tested only at two levels, low and high. Biosurfactant concentration (g/L) served as the only response variable. Fermentation medium was formulated according to the experimental design and based on 50 mL of each formulation dispensed into 250-mL Erlenmeyer flask. Phosphorus (1.0 g/L (NH_4_)_2_HPO_4_/NH_4_H_2_PO_4_) and carbon (5% v/v waste frying sunflower oil with composition of [% w/w] stearic acid [2.21], palmitic acid [6.11], volatile fractions (16.23), oleic acid [22.34], and linoleic acid [50.76]; saponification value 76 and density [30°C] 283.3 kg/m^3^) sources were added to the media, and flasks were corked and sterilized by autoclaving at 121°C for 15 min. Upon cooling, filter-sterilized urea (1.19 g/L) and inoculum (10% v/v-10^8^ cells/mL) were added to the medium, and flasks were incubated on a rotary shaker agitating at 150 rpm at room temperature for 72 h. All arrangements were made in triplicates.

Biosurfactant quantification was performed as described elsewhere[6]. Data were analyzed using the same statistical software that generated the design. Significant trace elements were selected for RSM and optimization.

### Response surface design and fermentation studies

The experimental design employed to fit the multiple regression models of the fermentation study was a 2^5-1^ half-fractional factorial central composite rotatable design. The test variables were Ni^2+^, Zn^2+^, Fe^3+^, B^3+^, and Cu^2+^; each at five levels. The selection of factor levels for RSM followed, on one hand, the method of the path of steepest ascent, a procedure that moves nutrient levels sequentially in the direction of maximum increase in the response investigated, and on the other, a consideration for the introduction of biomass concentration as a second response variable to facilitate the determination of biosurfactant production yield, Yp/x. Results of preliminary screening for the effects of increasing concentrations of metals on the growth of the bacterium were also considered in the selection of factor levels of significant trace nutrients from PBD for biomass and biosurfactant formation. Actual levels of each factor were calculated using the equation of Myers and Montgomery[20].


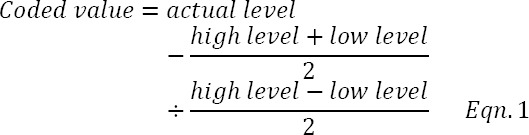


The coded levels were determined as follows: *X_1_*=(Ni^2+^-0.75)/0.25, *X_2_*=(Zn^2+^–0.075)/0.025, *X_3_*= (Fe^3+^–0.075)/0.025, *X_4_*=(B^3+^–0.03)/0.01, and *X*_5_=(Cu^2+^-0.075)/0.025. The response variables were biomass Y_1_ (g/L) and biosurfactant Y_2_ (g/L) concentrations. The experimental design required 32 experimental runs, which were set up in 250-mL Erlenmeyer flasks, each containing 50 mL of fermentation medium composed (g/L) of Na_2_HPO_4_/KH_2_PO_4_ (2:1; 4.5), MgSO_4_.7H_2_O (0.2), NaCl (0.5), CaCl_2_ (0.5), KCl (0.5), MoNa_2_O_4_.2H_2_O (0.05 mg/L), CoCl_2_.6H_2_O (0.05 mg/L), and MnSO_4_.4H_2_O (0.05 mg/L). Different concentrations of the five significant trace elements obtained from PBD screening were then added to the flasks according to the actual values of the factor levels specified by the coded values in Table 1. Medium pH was adjusted to 7.0 with KOH pellets[4]. Waste frying oil was subsequently added to each flask at 5% (v/v) concentration and flasks, without nitrogen source, were sterilized by autoclaving at 121°C for 15 min. Upon cooling, filter-sterilized urea (1.19 g/L) and inoculum (10% v/v–10^8^ cells/mL) were introduced into flasks prepared in triplicates, and then the flasks were incubated on rotary shakers agitating at 150 rpm at room temperature for 72 h.

**Table 1:** Placket-Burman design matrix (randomized) for trace element contribution to biosurfactant formation in coded units

Run	A	B	C	D	E	F	G	H	J	K	L	M	BSC (g/L)
1	-1	-1	-1	1	-1	1	-1	1	1	1	1	-1	23.58
2	1	1	1	1	-1	-1	1	1	-1	1	1	-1	25.96
3	-1	-1	1	1	-1	1	1	-1	-1	-1	-1	1	24.21
4	1	-1	-1	1	1	-1	1	1	-1	-1	-1	-1	21.43
5	-1	1	1	-1	-1	-1	-1	1	-1	1	-1	1	24.19
6	1	1	-1	1	1	-1	-1	-1	-1	1	-1	1	28.96
7	-1	1	1	1	1	-1	-1	1	1	-1	1	1	31.73
8	1	1	-1	-1	-1	-1	1	-1	1	-1	1	1	32.75
9	-1	-1	-1	-1	-1	-1	-1	-1	-1	-1	-1	-1	17.45
10	1	-1	1	-1	1	1	1	1	-1	-1	1	1	36.02
11	1	-1	1	1	1	1	-1	-1	1	1	-1	1	24.72
12	-1	1	-1	1	-1	1	1	1	1	-1	-1	1	23.48
13	1	1	1	-1	-1	1	1	-1	1	1	-1	-1	22.31
14	1	-1	1	1	-1	-1	-1	-1	1	-1	1	-1	27.08
15	-1	-1	-1	-1	1	-1	1	-1	1	1	1	1	33.27
16	-1	1	-1	1	1	1	1	-1	-1	1	1	-1	25.31
17	-1	1	1	-1	1	1	-1	-1	-1	-1	1	-1	28.78
18	1	1	-1	-1	1	1	-1	1	1	-1	-1	-1	21.08
19	1	-1	-1	-1	-1	1	-1	1	-1	1	1	1	33.10
20	-1	-1	1	-1	1	-1	1	1	1	1	-1	-1	23.29

A, boron; B, calcium, C, cobalt; D, copper; E, iron; F, potassium; G, magnesium; H, manganese; J, molybdenum; K, sodium; L, nickel; M, zinc; ‘1’, high value; ‘-1‘, low value; BSC, biosurfactant concentration

### Determination of biomass (Y1) and biosurfactant (Y2) concentrations

Small portions of 72-h fermentation broth from each experimental setting (10 mL) were centrifuged at 8,000 ×g for 10 min. Cell-free supernatants were collected, and the cell pellets were washed twice in de-mineralized water. The supernatants were subjected to 0.45-µM and 0.2-µM Millipore membrane filtrations and subsequently to acid precipitation with 6N HCl, pH 2.0. Biomass and biosurfactant concentrations were determined from cell pellets and acid precipitates of supernatants, respectively as described previously[6].

### Statistical analysis

All data generated from the factorial experiment were subjected to multiple regression analysis using least squares to build the regression models. A second-order (quadratic) function was used to fit the data generated. Experimental design, data analysis, interaction plotting, and optimization of factor conditions were done with MINITAB 17 statistical software, while Excel 2007 was used for confirmation of model fits where predicted responses were plotted against experimentally-derived data. All hypotheses were tested at 95% confidence level.

For the five factors considered in the optimization experiment, the quadratic model took the form below:


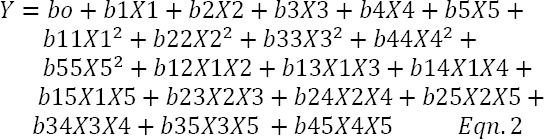


where *b*0 is the value of the fixed response at the central point (0, 0, 0, 0, 0); *b*1, *b*2, *b*3, *b*4, and *b*5 the coefficients of the linear terms; *b*11, *b*22, *b*33, *b*44, and *b*55 the coefficients of the quadratic terms; *b*12, *b*13, *b*14, b15, *b*23, *b*24, *b*25, *b*34, *b*35, and *b*45 are the coefficients of the cross products (interactive terms).

### Verification experiments

Studies to confirm the validity of the results of the optimization experiment were done by setting up glycolipopeptide production in the optimized medium. The medium had the same composition as that described in Materials and Methods with concentrations of nickel, zinc, iron, boron, and copper incorporated in accordance with the results of the various optimized conditions. Fermentation was conducted in 250-mL Erlenmeyer flasks containing 50 mL of fermentation medium supplemented with 5% (v/v) waste frying sunflower oil as carbon source. Cooled sterilized media were supplemented with filter-sterilized urea (1.19 g/L) and then inoculated with 10% (v/v–10^8^ cells/mL) inoculum of *Pseudomonas aeruginosa* strain IKW1. Flasks were incubated as earlier described and biomass and biosurfactant concentrations determined as described in Ekpenyong *et al*.[6]. Means of triplicate determinations of concentrations of biomass and biosurfactant from corroborating experiments were compared with those predicted by the regression models.

## RESULTS AND DISCUSSION

### Plackett-Burman design

The design matrix of the PBD for the effects of 12 trace nutrients on biosurfactant production and their responses are shown in Table 1. Results showed the highest biosurfactant concentration of 36.02 g/L in run 10. However, the results of the trace element modeling experiment by PBD revealed that only 5 out of 12 nutrient elements significantly influenced the glycolipopeptide production. The non-selection of the remaining seven elements suggests their non-significant (*P*>0.05) contributions to the response under investigation at the confidence level selected for the study.

**Fig. 1:**
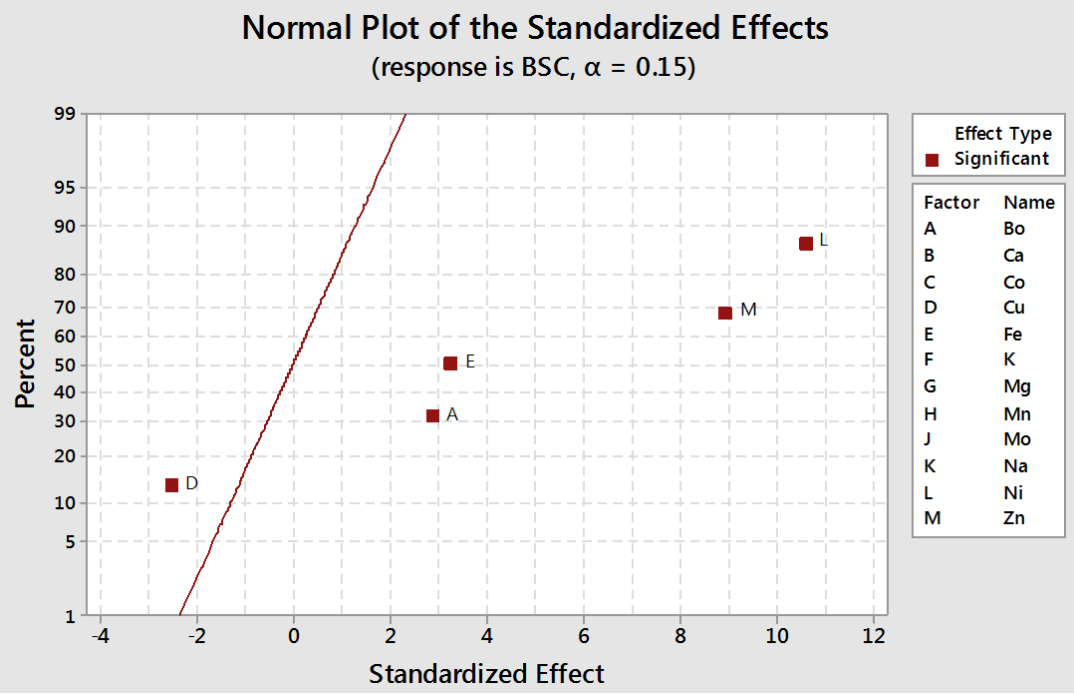
Normal plot of standardized effects of significant trace nutrients of a Plackett-Burman design for glycolipoeptide-biosurfactant production. Bo is used loosely to indicate boron and not as a chemical symbol.

**Fig. 2:**
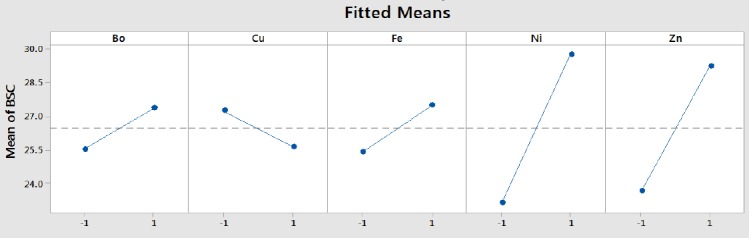
Main effects plots of contributions of significant trace elements to glycolipopeptide-biosurfactant production by *Pseudomonas aeruginosa* strain IKW1. BSC, biosurfactant concentration

Results of the trace element screen test by PBD indicated the same results as trace element modeling experiment. Figure 1 is the normal plot of the standardized effect of the significant nutrients, showing the magnitude and direction of their significant effects. The Figure reveals that nickel has the highest significant positive effect on biosurfactant production by the bacterium since its effect is positioned the furthest to the right of the response line. Other nutrients with significant enhancement effect on biosurfactant production were zinc, iron, and boron, which is in agreement with the Joshi *et al*.’s study[17]. However, the Figure reveals a significant reductive effect of copper on glycolipopeptide production by the bacterium since its effect is positioned to the left of the biosurfactant response line. Figure 2 shows the main effects plots of significant trace nutrients on the response variable and confirms the results displayed in Figure 1. Nickel has been shown to make the highest contribution to biosurfactant production, whereas copper exerts a negative effect on biosurfactant production.

The analysis of variance (ANOVA) result for significant trace elements is demonstrated in Table 2 and confirms nickel as having the most significant (*P*<0.05) enhancement effect on biosurfactant production given by its very large *F* value. The linear regression coefficient of determination, adjusted *R*^2^ of 91.81%, indicates that the model equation (below), given in un-coded units, was significant and could explain 91.81% of the variability in the response data. The equation reveals that nickel has the largest coefficient that is preceded by a positive sign, confirming once again its strong enhancement effect on biosurfactant formation.

**Table 2:** Analysis of variance (ANOVA) of the regression model from the Plackett-Burman design for trace element contribution to biosurfactant formation in un-coded units

Source	DF	Adj. SS	Adj. MS	*F* value	*P* value
Model	5	428.38	85.677	43.61	0.000
Linear	5	428.38	85.677	43.61	0.000
Bo	1	16.42	16.417	8.36	0.012
Cu	1	12.45	12.450	6.34	0.025
Fe	1	20.97	20.972	10.67	0.006
Ni	1	220.85	220.847	112.41	0.000
Zn	1	157.70	157.697	80.27	0.000
Error	14	27.51	1.965		
Total	19	455.89			

Model Summary: S, 1.40166; *R*^2^, 93.97%; adjusted *R*^2^, 91.81%; predicted *R*^2^, 87.69%; *P*<0.05, 5% significance level. Bo is used loosely to indicate boron and not as a chemical symbol. DF, degrees of freedom; SS, sum of squares; MS, mean sum of squares.


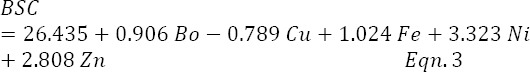


### Response surface optimization

#### Experimental data of the response surface method

Table 3 shows the actual factor levels corresponding to coded factor levels for a 2^5-1^ half-fractional factorial, a central composite rotatable design of RSM. The experimental responses from the 32 experimental runs of the surface methodology are presented in Table 4. Maximum biomass concentration of 21.07 g/L was obtained at conditions set at (X_1_, X_2_, X_3_, X_4_, X_5_)=(-1, -1, 1, -1, -1) corresponding to 0.5 mg/L nickel, 0.05 mg/L zinc, 0.1 mg/L iron, 0.02 mg/L boron, and 0.05 mg/L copper. In these conditions, biosurfactant concentration obtained was 29.56 g/L. However, the highest biosurfactant concentration of 57.21 g/L was acquired at conditions set at (X_1_, X_2_, X_3_, X_4_, X_5_)=(1, 1, 1, -1, -1) corresponding to 1 mg/L nickel, 0.1 mg/L zinc, 0.1 mg/L iron, 0.02 mg/L boron, and 0.05 mg/L copper and a corresponding biomass concentration of 19.53 g/L. Glycolipopeptide production yield, Yp/x, under these conditions would be 2.93. The attainment of peak biomass and biosurfactant concentrations at different experimental runs suggest the requirement of different trace nutrient conditions for cellular growth and metabolite synthesis, especially metabolites obtained during or near idiophasic metabolism of organisms.

**Table 3:** Actual factor levels corresponding to coded factor levels for 2^5-1^ half-fractional factorial central composite rotatable design of response surface method

Variable (mg/L)	Actual values					

	Code	-2	-1	0	1	2
NiCl_2_.6H_2_O	X_1_	0.250	0.50	0.750	1.00	1.250
ZnSO_4_.7H_2_O	X_2_	0.025	0.05	0.075	0.10	0.125
FeCl_3_	X_3_	0.025	0.05	0.075	0.10	0.125
K_3_BO_3_	X_4_	0.010	0.02	0.030	0.04	0.050
CuSO_4_.5H_2_O	X5	0.025	0.05	0.075	0.10	0.125

**Table 4:** Actual factor levels corresponding to coded factor levels for the CCRD of the response surface optimization showing biomass and biosurfactant concentrations

Run order	X1	X2	X3	X4	X5	NiCl_2_ (mg/L)	ZnSO_4_ (mg/L)	FeCl_3_ (mg/L)	K_3_BO_3_ (mg/L)	CuSO_4_ (mg/L)	BMC (g/L)	BSC (g/L)
1	0	0	0	0	0	0.750	0.075	0.075	0.030	0.075	16.86	41.03
2	-2	0	0	0	0	0.250	0.075	0.075	0.030	0.075	14.36	29.55
3	-1	-1	-1	1	-1	0.500	0.050	0.050	0.040	0.050	8.96	36.04
4	1	-1	1	1	-1	1.000	0.050	0.100	0.040	0.050	12.98	38.32
5	0	0	0	2	0	0.750	0.075	0.075	0.050	0.075	13.52	28.39
6	0	0	0	-2	0	0.750	0.075	0.075	0.010	0.075	18.52	36.57
7	0	0	0	0	-2	0.750	0.075	0.075	0.030	0.025	15.39	46.47
8	-1	1	-1	-1	-1	0.500	0.100	0.050	0.020	0.050	11.35	38.32
9	-1	1	1	1	-1	0.500	0.100	0.100	0.040	0.050	18.26	35.31
10	1	-1	-1	-1	-1	1.000	0.050	0.050	0.020	0.050	10.62	32.19
11	0	0	0	0	0	0.750	0.075	0.075	0.030	0.075	18.76	41.22
12	0	-2	0	0	0	0.750	0.025	0.075	0.030	0.075	9.67	36.82
13	0	0	0	0	0	0.750	0.075	0.075	0.030	0.075	18.87	41.46
14	-1	1	-1	1	1	0.500	0.100	0.050	0.040	0.100	13.28	27.54
15	-1	-1	1	1	1	0.500	0.050	0.100	0.040	0.100	13.41	26.79
16	0	0	2	0	0	0.750	0.075	0.125	0.030	0.075	16.59	40.45
17	1	1	-1	1	-1	1.000	0.100	0.050	0.040	0.050	12.38	31.07
18	1	1	-1	-1	1	1.000	0.100	0.050	0.020	0.100	16.58	30.01
19	0	0	-2	0	0	0.750	0.075	0.025	0.030	0.075	8.95	28.12
20	0	2	0	0	0	0.750	0.125	0.075	0.030	0.075	15.38	46.05
21	1	1	1	1	1	1.000	0.100	0.100	0.040	0.100	8.47	39.67
22	0	0	0	0	2	0.750	0.075	0.075	0.030	0.125	11.04	37.58
23	1	-1	1	-1	1	1.000	0.050	0.100	0.020	0.100	11.94	41.40
24	-1	-1	1	-1	-1	0.500	0.050	0.100	0.020	0.050	21.07	29.56
25	1	1	1	-1	-1	1.000	0.100	0.100	0.020	0.050	19.53	57.21
26	0	0	0	0	0	0.750	0.075	0.075	0.030	0.075	18.94	42.08
27	-1	1	1	-1	1	0.500	0.100	0.100	0.020	0.100	17.39	37.99
28	0	0	0	0	0	0.750	0.075	0.075	0.030	0.075	19.04	41.68
29	-1	-1	-1	-1	1	0.500	0.050	0.050	0.020	0.100	9.47	31.07
30	0	0	0	0	0	0.750	0.075	0.075	0.030	0.075	18.83	40.78
31	1	-1	-1	1	1	1.000	0.050	0.050	0.040	0.100	9.57	30.24
32	2	0	0	0	0	1.250	0.075	0.075	0.030	0.075	12.35	39.00

X1, nickel; X2, zinc; X3, iron; X4, boron; X5, copper; BMC, biomass concentration; BSC, biosurfactant concentration

### Regression model for biomass concentration, Y_1_

The data presented in Table 4 was subjected to multiple regression analyses using least squares regression to fit a second-order (quadratic) regression model for biomass concentration, Y_1_. The model stipulated 20 predictors; however, the result of ANOVA of the model in Table 5 reveals that 5 interactive terms were removed by stepwise selection, since their contributions were not significant at *P*=0.05. The *T* values of the predictor coefficients (data not shown) suggest that all model predictors, except the linear terms of zinc and iron, made significant negative contributions to the model, implying their reductive effects on biomass formation. The model equation is presented in un-coded units as equation 4 below:

**Table 5:** Analysis of variance of the 2^5-1^ half-fractional factorial central composite rotatable design of an response surface method for biomass regression model in un-coded units

Source	DF	Adjusted SS	Adjusted MS	*F* value	*P* value
Model	15	435.332	29.0222	35.12	0.000
Linear	5	199.895	39.9790	48.38	0.000
Ni	1	9.551	9.5508	11.56	0.004
Zn	1	39.117	39.1171	47.34	0.000
Fe	1	88.627	88.6273	107.26	0.000
Bo	1	39.117	39.1171	47.34	0.000
Cu	1	23.483	23.4828	28.42	0.000
Square	5	134.701	26.9401	32.60	0.000
Ni*Ni	1	35.179	35.1787	42.57	0.000
Zn*Zn	1	49.773	49.7729	60.23	0.000
Fe*Fe	1	45.202	45.2022	54.70	0.000
Bo*Bo	1	5.395	5.3951	6.53	0.021
Cu*Cu	1	37.463	37.4633	45.34	0.000
Two-way interaction	5	100.737	20.1473	24.38	0.000
Ni*Fe	1	33.931	33.9306	41.06	0.000
Ni*Bo	1	6.126	6.1256	7.41	0.015
Zn*Fe	1	7.182	7.1824	8.69	0.009
Fe*Bo	1	10.530	10.5300	12.74	0.003
Fe*Cu	1	42.968	42.9680	52.00	0.000
Error	16	13.221	0.8263		
Lack-of-fit	11	9.748	0.8862	1.28	0.418
Pure error	5	3.473	0.6946		
Total	31	448.554			

Model Summary: S, 0.909022; R^2^, 97.05%; adjusted R^2^, 94.29%; Predicted R^2^, 86.75%; *P*<0.05, 5% significance level.Bo is used loosely to indicate boron and not as a chemical symbol. DF, degrees of freedom; SS, sum of squares; MS, mean sum of squares


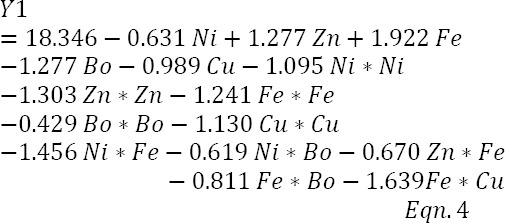


The metal, iron, is described as the only macro-bio-element of the heavy metals and the most biologically relevant trace nutrient[18]. The effect of its linear term on biomass formation was understandably pronounced, and the significant reductive effect of its quadratic term suggests possible toxicity at high concentrations and therefore its trace requirement. Abalos *et al*.[14] have reported a similar result trend during production of rhamnolipid from a strain of *Pseudomonas aeruginosa*.

The requirement of zinc for biomass formation derives from its role in DNA-binding proteins and a variety of enzymes. Zinc is also known to act as a Lewis base needed to activate water for involvement in aqueous reactions[6].

The significance of a model is given by its goodness-of-fit test, often expressed as the coefficient of determination *R*^2^, which is the percentage of the variations in the response that can be explained by independent factors and their interactions. In this study, the biomass regression model (Table 5) was highly significant (*F*=35.12; *P*=0.000) with an adjusted *R*^2^ of 94.29%, indicating that only 5.71% of the variability in the response is not explainable by the model.

A well-fitted model estimated by adjusted *R*^2^ might not adequately explain data variations in the region of experimentation. The lack-of-fit test is therefore frequently used as a support test for adequacy of the fitted model. The ANOVA table for the biomass regression model in this study shows a non-significant lack-of-fit (*F*=1.28; *P*=0.418) for the model, which suggests its adequacy for the explanation of data variations in the region of experimentation.

**Fig. 3:**
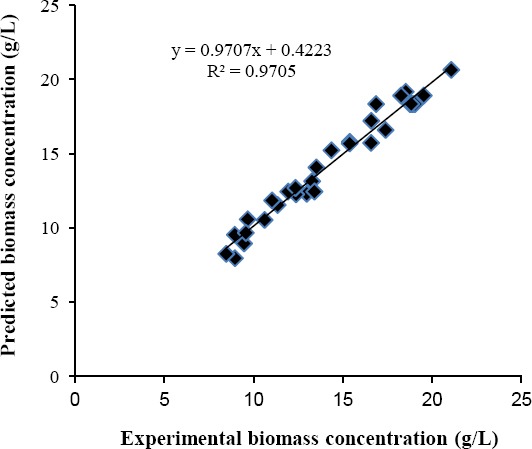
Experimental biomass concentration plotted against biomass concentration predicted by the fitted model.

The regression plot of experimental against predicted biomass responses is presented as Figure 3 and reveals a coefficient of determination, *R*^2^ of 0.9705, which is in agreement with the *R*^2^ for the model in the ANOVA table.

### Regression model for biosurfactant concentration, Y_2_

The ANOVA for the biosurfactant regression model presented in Table 6 indicated that the model was highly significant (*F*=371.07; *P*=0.000). All the linear predictor terms and only the square terms of nickel, iron, and boron were significant. Six of the interactive predictor terms were also significant; however, Fe*Cu interaction, although included in the model, was not significant (*P*=0.051). The magnitude and direction of significant effect of model predictors are given by the *T* values of the coefficient estimates of the predictors (data not shown) and reveal that the linear, but not the quadratic terms of nickel, zinc, and iron, significantly enhanced biosurfactant formation. It is probably because of the enhancing effect of Fe*Cu that the term was included in the model, to increase biosurfactant concentration as long as the adjusted *R*^2^ of the model was not negatively affected. The model equation, in un-coded units, is therefore presented as equation 5 below.

**Table 6:** Analysis of variance of the 2^5-1^ half fractional factorial central composite rotatable design (CCRD) of an response surface method for biosurfactant regression model in un-coded units

Source	DF	Adjusted SS	Adjusted MS	*F* value	*P* value
Model	15	1388.26	92.551	371.07	0.000
Linear	5	676.69	135.338	542.62	0.000
Ni	1	132.49	132.493	531.21	0.000
Zn	1	104.04	104.042	417.14	0.000
Fe	1	230.83	230.826	925.46	0.000
Bo	1	100.57	100.573	403.23	0.000
Cu	1	108.76	108.758	436.05	0.000
Square	3	326.18	108.726	435.92	0.000
Ni*Ni	1	105.31	105.312	422.24	0.000
Fe*Fe	1	105.03	103.033	421.11	0.000
Bo*Bo	1	161.50	161.502	647.52	0.000
2-Way Interaction	7	385.39	55.056	220.74	0.000
Ni*Fe	1	198.88	198.881	797.38	0.000
Ni*Bo	1	6.57	6.566	26.33	0.000
Zn*Fe	1	84.23	84.227	337.69	0.000
Zn*Bo	1	45.93	45.935	184.17	0.000
Zn*Cu	1	25.23	25.226	101.14	0.000
Fe*Bo	1	23.45	23.450	94.02	0.000
Fe*Cu	1	1.11	1.108	4.44	0.051
Error	16	3.99	0.249		
Lack-of-Fit	11	2.90	0.263	1.20	0.446
Pure Error	5	1.09	0.219		
Total	31	1392.25			

Model Summary: S, 0.499416; *R^2^*, 99.71%; adjusted *R^2^*, 99.44%; predicted *R^2^*, 98.77%; *P*<0.05, 5% significance level. Bo is used loosely to indicate boron and not as a chemical symbol. DF, degrees of freedom; SS, sum of squares; MS, mean sum of squares.


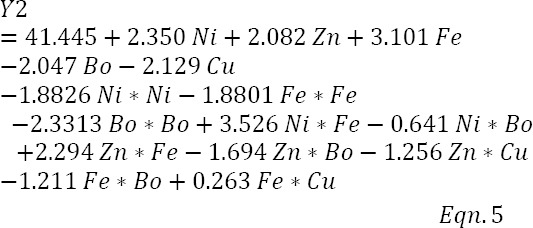


The model was shown to have an adjusted *R*^2^ value of 99.44%, suggesting that only 0.56% of the variability in biosurfactant responses could not be explained by it, and that the observed variations were due to the factor effects and not due to noise. To confirm the adequacy of the biosurfactant model in explaining the variations about the data, an examination of the lack-of-fit test result in the ANOVA table was made and demonstrated a non-significant (*F*=1.20; *P*=0.446) lack-of-fit, indicating the adequacy of the model to explain data in the experimental region.

**Fig. 4:**
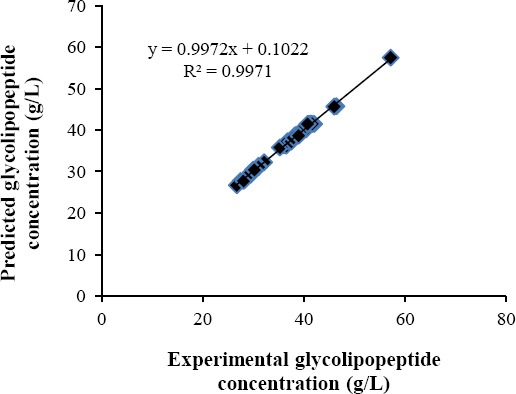
Experimental biosurfactant concentrations versus theoretical values predicted by the regression model.

A plot of experimental biosurfactant concentrations against predicted concentrations obtained by solving equation 5 is presented in Figure 4 as the final ratification of the fit of the biosurfactant regression model and confirms a goodness-of-fit, *R*^2^ (unadjusted) value of 99.97%.

### Contour and surface plots for biomass (Y1) and biosurfactant (Y2) concentrations

**Fig. 5:**
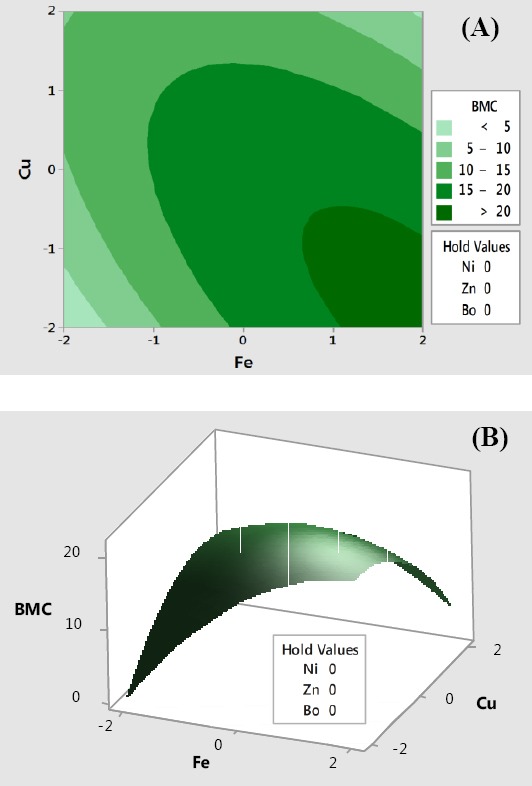
Contour (A) and surface (B) plots of two-way interactions of independent variables for maximal biomass production. Bo is used loosely to indicate boron and not as a chemical symbol. BMC, biomass concentration

Biomass response plots were made with the vertical axis representing biomass concentration (Y1) and two horizontal axes representing the most significant two- way interaction (X3, X5)=(Fe, Cu). The plots led to maximal biomass formation with the remaining three factors (X1, X2, X4)=(Ni, Zn, Bo) held at their optimum levels. Biomass concentration under this condition was in excess of 20 g/L. The contour and surface plots displayed in Figure 5 reveal that maximal biomass would be accumulated under the high levels of iron and low levels of copper.

**Fig. 6:**
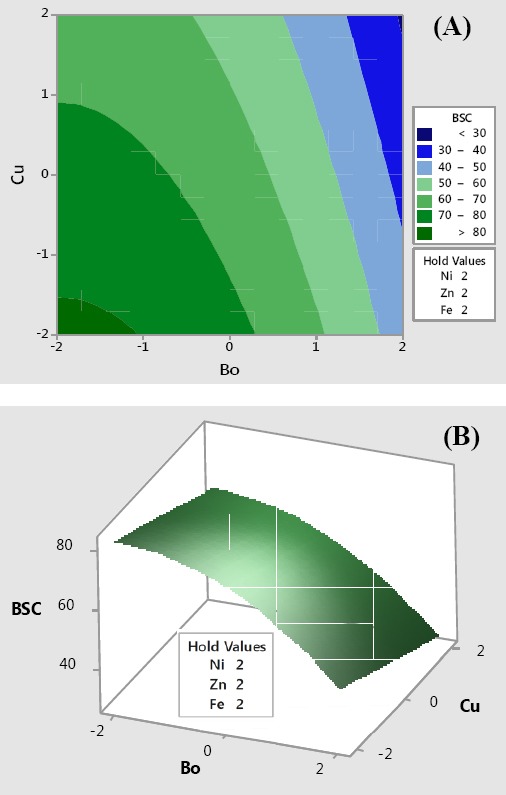
Contour (A) and surface (B) plots of two-way interactions of independent variables for maximal glycolipopeptide production. Bo is used loosely to indicate boron and not as a chemical symbol. BSC, biosurfactant concentration

The biosurfactant response plots (Fig. 6) were made with the vertical axis representing biosurfactant concentration (Y2) and two horizontal axes representing the two most significant variables (X4, X5)=(Bo, Cu) that led to maximal biosurfactant formation with the remaining three factors (X1, X2, X3)=(Ni, Zn, Fe) held at their optimum levels. Maximal biosurfactant concentration under this condition was in excess of 80 g/L. The contour and surface plots reveal that the highest biosurfactant concentration would be obtained when fermentation medium contains low levels of both copper and boron, with the concentrations of nickel, zinc, and iron supplied at high levels.

Boron, a micro-nutrient with intermediary properties of metals and non-metals, is known as a regulator of certain pathways that require serine proteases or oxidoreductases involving co-enzymes like nicotinamide adenine dinucleotide and nicotinamide adenine dinucleotide phosphate for activity[22]. These activities of boron in bacteria could very well be the reason for its very little requirement both for cellular growth and secondary metabolism.

Copper, on the other hand, is mostly important in cytochrome C oxidases; oxygen-dependent terminal oxidases in the electron transport chain of aerobic organisms[23]. The interaction therefore between boron and copper has to be very important in biosurfactant synthesis since the whole process is energy driven; copper to generate energy and boron to inhibit the process.

Nickel is a transition metal useful only in a few selected reactions. It associates with iron in NiFe hydrogenases; an enzyme system that splits molecular hydrogen into protons and electrons. The metal is also intrinsically bound to urease; an enzyme that catalyses the splitting of urea into carbon dioxide and ammonia thus supplying nitrogen in its available form for cellular metabolism[18,24]. The preference of urea as a nitrogen source for glycolipopeptide biosurfactant production by *Pseudomonas aeruginosa* strain IKW1 (unpublished data) demystifies the significant enhancement effect of nickel on biosurfactant production and suggests a correlation between urease activity and biosurfactant production in the bacterium.

### Selection of optimum conditions for response variables

For situations where biomass accumulation is desired instead of the surface-active compound, which was not the case in this study, the highest predictable biomass concentration of 26.55 g/L was obtained at trace mineral conditions set at (X1, X2, X3, X4, X5)=(-1.0707, -0.0202, 2.0, -2.0, -1.8788) corresponding to 0.482 mg/L nickel, 0.074 mg/L zinc, 0.125 mg/L iron, 0.010 mg/L boron, and 0.028 mg/L copper.

For tertiary oil recovery and spilled oil remediation where maximum concentrations of both responses are desired, trace mineral conditions set at (X1, X2, X3, X4, X5)=(0.2626, 0.9495, 2.0, -2.0, -2.0) corresponding to 0.816 mg/L nickel, 0.099 mg/L zinc, 0.125 mg/L iron, 0.01 mg/L boron, and 0.025 mg/L copper, which would lead to 23.4 g/L biomass and 57.55 g/L biosurfactant.

Finally, for detergent, food, cosmetic, and pharmaceutical applications where maximum surface-active compound and zero microbial cells are desired, the response optimizer set conditions for maximum biosurfactant concentration of 81.92 g/L at (X1, X2, X3, X4, X5)=(2.0, 2.0, 2.0, -1.9596, -2) corresponding to 1.25 mg/L nickel, 0.125 mg/L zinc, 0.125 mg/L iron, 0.0104 mg/L boron, and 0.025 mg/L copper.

### Verification experiments

Table 7 shows that maximum biosurfactant concentration obtained in the validation experiments under conditions (X1, X2, X3, X4, X5) =(2, 2, 2, -1.9596, -2) corresponding to 1.25 mg/L nickel, 0.125 mg/L zinc, 0.125 mg/L iron, 0.0104 mg/L boron, and 0.025 mg/L copper was 84.44 g/L with a corresponding biomass concentration of 19.14 g/L, giving a production yield (Y_p/x_) of 4.41. When fermentation conditions were set to maximize both responses, biosurfactant yield of 2.26 was achieved. Compared to the 81.92 g/L biosurfactant concentration predicted by the optimizer, the concentration obtained in the confirmation experiment was only 0.03% higher, thus validating the prediction of the response optimizer. A glycolipopeptide biosurfactant concentration of 84.44 g/L, when compared to 23.86 g/L obtained from a previous (control) experiment (unpublished data), reveals a ~3.54fold increase in biosurfactant concentration, thereby making separate optimization experiments for trace nutrients appropriate for developing the fermentation media for the production of microbial metabolites.

**Table 7:** Design codes, actual values, experimental and predicted responses of validation experiments of an RSM for glycolipopeptide production

Parameters	BMC maximized	BSC maximized	BMC and BSC maximized
X1	-1.0707	2	0.2626
X2	-0.0202	2	0.9495
X3	2	2	2
X4	-2	-1.9596	-2
X5	-1.8788	-2	-2
Ni (mg/L)	0.482	1.25	0.816
Zn (mg/L)	0.074	0.125	0.099
Fe (mg/L)	0.125	0.125	0.125
Bo (mg/L)	0.010	0.010	0.010
Cu (mg/L)	0.028	0.025	0.025
eBMC (g/L	25.74	19.14	25.14
pBMC (g/L)	26.55	NP	23.40
eBSC (g/L)	25.96	84.44	56.83
pBSC (g/L)	NP	81.92	57.55

X_1_, nickel (Ni); X_2_, zinc (Zn); X_3_, iron (Fe); X_4_, boron (Bo); X5, copper (Cu); eBMC, experimental biomass concentration; pBMC, predicted biomass concentration; eBSC, experimental biosurfactant concentration; pBSC, predicted biosurfactant concentration; NP, not predicted. Bo is used loosely to indicate boron and not as a chemical symbol.

PBD selected nickel, zinc, iron, boron, and copper as the most significant (*P*<0.05; adjusted *R*^2^=91.82%) trace minerals for glycolipopeptide biosurfactant production. Optimization of the nutrients by RSM resulted in 84.44 g/L of the biosurfactant under conditions set at (X1, X2, X3, X4, X5)=(2, 2, 2, -1.9596, -2). These conditions corresponded to 1.2500 mg/L nickel, 0.125 mg/L zinc, 0.125 mg/L iron, 0.0104 mg/L boron, and 0.025 mg/L copper, giving a production yield, Yp/x of 4.41. In conclusion, PBD and RSM are dependable tools for selecting and optimizing conditions of nutrients for biosurfactant production.
